# Bellows stiffness characteristics of cord-reinforced air spring with winding formation under preload conditions

**DOI:** 10.1038/s41598-023-29474-3

**Published:** 2023-02-28

**Authors:** Yu-Qiang Cheng, Hua Gao, Chang-Geng Shuai

**Affiliations:** 1 Institute of Noise and Vibration, Naval University of Engineering, Wuhan, 430033 China; 2 State Key Lab of Ship Vibration and Noise, Wuhan, 430033 China

**Keywords:** Mathematics and computing, Mechanical engineering, Composites

## Abstract

The bellows structure in an air spring can be constantly reinforced to cope with the complicated work environment, but it exerts a stronger and stronger effect on the stiffness characteristics of the air spring. However, there is not any effective way for the parameterized solution of the bellows stiffness of the air spring. With the precise transfer matrix method, the bellows stiffness characteristics of a cord-reinforced air spring with winding formation under preload conditions were analyzed in this paper. The thin-shell theory was used to solve the bellows pre-stress of the air spring under preload conditions. The pre-stress was introduced into the equilibrium equation for the bellows. Based on the geometrical and physical equations for the bellows with the complex cord winding characteristics, the precise integration method was borrowed to construct a transfer matrix for the bellows of the air spring under preload conditions. The state vector of the bellows in the air spring was solved through boundary conditions. The iteration method was adopted to develop the expression for the bellows stiffness characteristics, and combined with the theoretical model of pneumatic stiffness to solve the stiffness characteristics of the air spring. The comparison with the prototype test results verified the validity and correctness of the theoretical model. On this basis, we explored the influence of preload conditions, geometrical structure, and material characteristics on the stiffness characteristics of the air spring. The research findings will provide significant guidance for the structural design and material selection of cord-reinforced air springs with winding formation.

## Introduction

An air spring relies on the stiffness and damping characteristics of the compressed air to isolate the vibration and impact of equipment. As a vibration isolator, it has been widely applied in vehicles and vessels for vibration and noise reduction^1–5^. Compared with vehicles, a vessel offers a limited space for the installation of an air spring and requires greater bearing capacity. Hence, the air spring for vibration isolation in a vessel must be characterized by small size and large load. The operating air pressure of an air spring in a vessel is often greater than that of an air spring in general applications. In order to guarantee the reliability of an air spring under high internal pressure or other harsh environments, the cord skeleton layer of its bellows must be made of the cord of higher strength, and contains more layers of cord than ordinary air springs.

It is commonly believed that the vertical stiffness of an air spring depends on the reaction force generated by the compressed air contained in the air spring during its vertical deformation. The bellows exerts a little effect on the vertical stiffness characteristics of the air spring. In a simplified analytical model for the stiffness characteristics of an air spring, the effect of its bellows has been even ignored^6,7^. An air spring is facing the increasing demand for its reliability, so its bellows structure has to be constantly reinforced, exerting a stronger effect on the stiffness characteristics of the air spring. Therefore, the bellows stiffness should not be ignored anymore, and may even become a predominant component of stiffness characteristics after surpassing the pneumatic stiffness.

The bellows of an air spring is made of rubber matrix cord-reinforced composites. It is very complicated to construct its mechanical model because of its anisotropy. For this reason, the studies have mainly focused on pneumatic stiffness in the mechanical model for air spring parameterization^8–11^. The mechanical model for the bellows of an air spring is often analyzed using an equivalent model or a finite element model. There is not yet an effective parameterized model built for the bellows of an air spring. For instance, Erin and Wilson analyzed the stiffness characteristics of an air spring by simulating the nonlinear characteristics of its bellows through the parallel connection of a linear spring, a damper and a hysteretic damper^12^. Chen et al. put forward an air spring stiffness model comprising a structural parameter prediction model and a rubber bellow model. The rubber bellow model was a nonlinear equivalent model formed by a fractional Kelvin–Voigt model and a smooth friction model^13^. Zhu et al. constructed a universal air spring stiffness model after taking into account the contribution of internal pneumatic thermodynamics and the rubber friction and visco-elastic effects of the bellow rubber. While keeping the smooth friction model developed by Berg, they obtained the standard deviation of displacement excitation through statistics, and then determined the parameters of the friction model^14–16^. Shi et al.^17^ built an air spring finite element model, and adopted the sensitivity analysis method to explore the influence of geometrical parameters on the stiffness characteristics of the air spring. Wong et al.^18^ utilized the software ABAQUS to describe the nonlinear characteristics of the bellows in the rebar section, and constructed a finite element model for the air spring. On this basis, they analyzed how the mechanical performance of an air spring was affected by cord winding angle, effective radius and initial internal pressure.

An air spring is generally deformed under the joint effect of the internal pressure and the external loads. While constructing a mechanical model for the rubber matrix cord-reinforced bellows, attention should be paid to the complex anisotropic characteristics of cord-reinforced materials. It is also necessary to consider how the mechanical model for the bellows is affected by the pre-stress of the bellows under preload conditions and a strong coupling between the bellows state vector and the internal pressure of the air spring during deformation. All these factors bring a challenge to the construction of a parameterized model for the bellows of the air spring.

With the precise transfer matrix method, a parameterized model was built in this paper for the bellows stiffness of a cord-reinforced air spring under preload conditions. The thin-shell theory was first borrowed to solve the pre-stress of the bellows in the air spring under the effect of preload. Then the equilibrium equation for the bellows was recreated with the pre-stress. Based on the geometrical and physical equations for the bellows with the complex filament winding characteristics, the precise integration method was borrowed to construct a transfer matrix for the bellows of the cord-reinforced air spring with winding formation under preload conditions. In the end, the boundary conditions were taken into account to solve the state vector of the bellows. The iteration method was adopted to identify the coupling relationship between the bellows state vector and the internal pressure. The existing pneumatic stiffness model for an air spring was then used to determine the stiffness characteristics of the air spring.

## Theoretical modeling and solving

### Building a mechanical model for an air spring under preload conditions

**Figure 1:**
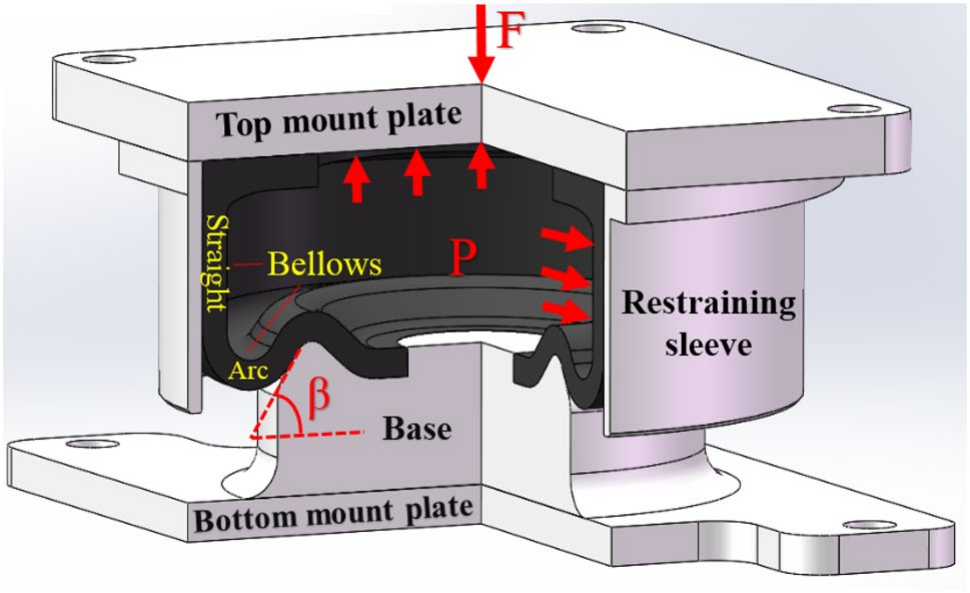
Schematic diagram of an air spring structure.

The structure of a cord-reinforced air spring with winding formation is given in Fig. 1. It contains a top mount plate, a bottom mount plate, a base, a rubber bellows, and a restraining sleeve. The restraining sleeve contacts the straight segment of the rubber bellows. During operation, the air spring can bear the equipment load* F* with the high-pressure *P* of the compressed air it contains, and the inner cavity of air spring is closed as well as the total amount of air is constant. In this case, the straight segment of the bellows is pressed against the restraining sleeve under high pressure. When the top mount plate of the air spring vibrates with the equipment, the arc segment of the bellows also curls and is deformed along the base. Consequently, the bellows stiffness of the air spring depends mainly on the mechanical state of the arc segment of the bellows in the process of deformation.

**Figure 2:**
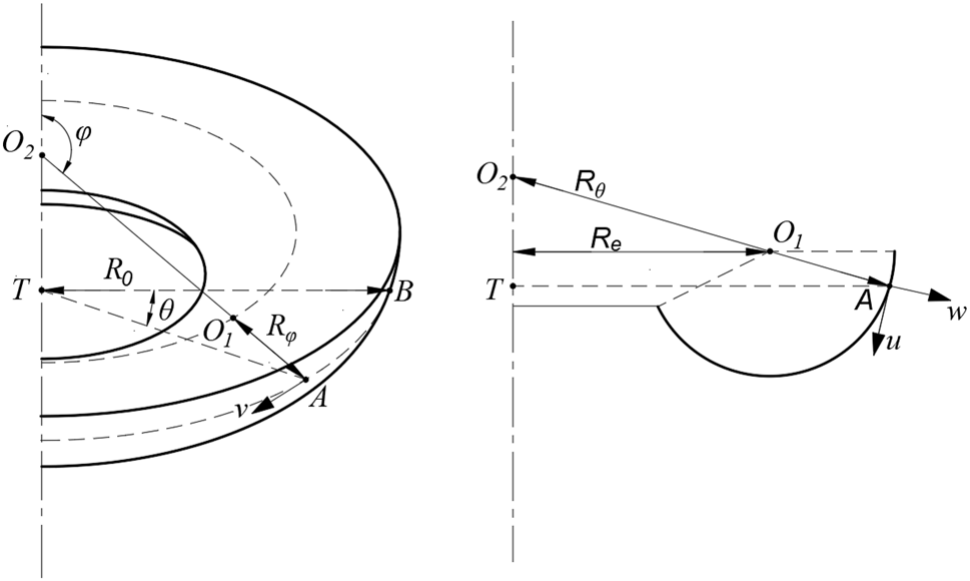
Schematic diagram of a rotary shell structure.

The arc segment of the bellows in the air spring is simplified into a rotary shell structure as illustrated in Fig. 2. The rotary shell is formed by a plane curve rotating around a coplanar axis. The curve is the meridian, and its plane is the meridian plane. The curve coordinates of any random point on the rotary shell are represented by (*φ*, *θ*) with *φ* as the meridian direction and *θ* as the longitudinal direction. The corresponding principal radii of curvature are indicated by *R*_*φ*_ and *R*_*θ*_, respectively. The radius of curvature of the latitudinal plane is denoted by *R*_*0*_. The lame coefficients of the rotary shell are *R*_*0*_ and *R*_*φ*_, respectively. Based on the relationship of the geometrical structure, we obtain:

1 $$ \left\{ {\begin{array}{*{20}l} {R_{\theta } = R_{\varphi } + \frac{{R_{e} }}{\sin \varphi }(\pi /2 \le \varphi \le \pi )} \hfill \\ {R_{\theta } = - R_{\varphi } - \frac{{R_{e} }}{\sin \varphi }(\pi < \varphi \le \pi + \beta )} \hfill \\ {R_{0} = R_{\theta } \sin \theta } \hfill \\ \end{array} } \right. $$

where *R*_*e*_ is the distance from the center of the curled bellows to the central axis, and *β* is the guiding angle of the base.

It is assumed that the preload force of the air spring is denoted by *F* during operation, and its internal pressure is denoted by *P*. Their relationship is defined by the following function^10^:

2 $$ F = P\pi R_{e}^{2} $$

The pre-stress on the section of unit length in the longitudel and latitudinal directions of is represented by *N*_*φ*0_ and *N*_*θ*0_, respectively. Based on the analysis with the thin-shell theory^19^, the pre-stress on the bellows in the air spring is expressed as:

3 $$ \left\{ {\begin{array}{*{20}l} {N_{\varphi 0} = P\left( {\frac{{R_{\varphi }^{2} sin(\varphi ) + 2R_{\varphi } R_{e} }}{{2R_{\varphi }^{{}} \sin (\varphi ) + 2R{}_{e}}}} \right)} \hfill \\ {N_{\theta 0} = P\frac{{R_{\varphi } }}{2}} \hfill \\ \end{array} } \right. $$

The mechanical model for the bellows of the cord-reinforced air spring involves geometrical, physical and equilibrium equations. Among them, the geometrical equation reflects the structural characteristics of the bellows, while the physical equation defines the material characteristics of the bellows. In this paper, the air spring is made by cord winding to further improve the strength of its bellows for application in the marine environment. Therefore, the cord winding angle varies at different positions of the bellows in the air spring, making it much more complicated to construct a mechanical model for the bellows. Based on the non-geodesic winding model and the laminate theoretical model are combined to create the physical equations for the bellows of the air spring with variable cord winding characteristics^20^.

In order to determine the stiffness characteristics of the air spring, the pre-stress in the bellows of the air spring must be considered under preload conditions while creating the equilibrium equation for the bellows. If the three-order slight amount and two-order disturbance quantity are ignored, the following equilibrium equation is derived on the basis of the flügge theory^21^ under the preload conditions:

4 $$ \left\{ {\begin{array}{*{20}l} {\frac{{\partial \left( {R_{0} N_{\varphi } } \right)}}{\partial \varphi } + R_{\varphi } \frac{{\partial N_{\theta \varphi } }}{\partial \theta } - R_{\varphi } \cos \varphi N_{\theta } + R_{0} Q_{\varphi } + \frac{{N_{{\varphi_{0} }} R_{0} \left( {\frac{{\partial^{2} u}}{{\partial \varphi^{2} }} + \frac{\partial \omega }{{\partial \varphi }}} \right)}}{{R_{\varphi } }} + \frac{{N_{{\theta_{0} }} }}{{R_{0} }}\frac{{\partial^{2} \mu }}{{\partial \theta^{2} }} - PR_{0} \left( {\frac{\partial \omega }{{\partial \varphi }}} \right) = 0} \hfill \\ {\frac{{\partial \left( {R_{0} N_{\varphi \theta } } \right)}}{\partial \varphi } + R_{\varphi } \frac{{\partial N_{\theta } }}{\partial \theta } + R_{\varphi } \cos \varphi N_{\theta \varphi } + R_{\varphi } \sin \varphi Q_{\theta } + \frac{{R_{\varphi } \left( {\frac{\partial u}{{\partial \theta }}\cos \varphi + \frac{{\partial^{2} v}}{{\partial \theta^{2} }} + \frac{\partial \omega }{{\partial \theta }}sin\varphi } \right)N_{{\theta_{0} }} }}{{R_{0} }} + N_{{\varphi_{0} }} R_{0} \frac{{\partial^{2} v}}{{\partial \theta^{2} }} = 0} \hfill \\ {\frac{{\partial \left( {R_{0} Q_{\varphi } } \right)}}{\partial \varphi } + R_{\varphi } \frac{{\partial Q_{\theta } }}{\partial \theta } - R_{\varphi } R_{0} \left( {\frac{{N_{\varphi } }}{{R_{\varphi } }} + \frac{{N_{\theta } }}{{R_{\theta } }}} \right) - \frac{{N_{{\theta_{0} }} R_{\varphi } \sin \varphi \left( {ucos\varphi + 2\frac{d}{\partial \theta }v(\theta ) + wsin\varphi - \frac{{\partial^{2} w}}{{\partial \theta^{2} }}} \right)}}{{R_{0} }}} \hfill \\ { + R_{0} N_{{\varphi_{0} }} \left( {\frac{{\partial^{2} w}}{{\partial^{2} \varphi }}} \right) + R_{\varphi } P\left( {\frac{\partial u}{{\partial \varphi }} + \frac{\partial v}{{\partial \theta }} + w} \right) = 0} \hfill \\ {Q_{\varphi } = \frac{1}{{R_{\varphi } R_{0} }}\left( {\frac{{\partial \left( {R_{0} M_{\varphi } } \right)}}{\partial \varphi } + R_{\varphi } \frac{{\partial M_{\theta \varphi } }}{\partial \theta } - R_{\varphi } \cos \varphi M_{\theta } } \right)} \hfill \\ {Q_{\theta } = \frac{1}{{R_{\varphi } R_{0} }}\left( {\frac{{\partial \left( {R_{0} M_{\varphi \theta } } \right)}}{\partial \varphi } + R_{\varphi } \frac{{\partial M_{\theta } }}{\partial \theta } + R_{\varphi } \cos \varphi M_{\theta \varphi } } \right)} \hfill \\ \end{array} } \right. $$

where *u*, *v* and *w* are the longitudinal, latitudinal, and normal displacement of the bellows; *θ*_*φ*_ is the curvature component; *M*_*φ*_ is the bending moment component; *h* is the thickness of cord layer; *Q*_*φ*_ and *Q*_*θ*_ are the amplitude of transverse shear on a unit length; *S*_*a*_ and *V*_*a*_ are the in-plane shear and transverse shear of the Kelvin-Kirchhoff model, and their amplitudes are defined by:

5 $$ S_{\varphi } = N_{\varphi \theta } + \frac{{M_{\varphi \theta } \sin \varphi }}{{R_{\varphi } }},V_{\varphi } = Q_{\varphi } + \frac{1}{{R_{\varphi } }}\frac{{\partial M_{\varphi \theta } }}{\partial \theta } $$

### Deriving a transfer matrix for the state vector of the bellows in an air spring

It is assumed that the state vector of the bellows in the air spring is *Z(φ)*, and contains eight state quantities defined by the following equation:

6 $$ Z(\varphi ) = \begin{array}{*{20}c} {\begin{array}{*{20}c} {\begin{array}{*{20}c} {\begin{array}{*{20}c} {[u(\varphi )} & {v(\varphi )} \\ \end{array} } & {w(\varphi )} & {\theta_{\varphi } (\varphi )} \\ \end{array} } & {N_{\varphi } (\varphi )} & {S_{\varphi } (\varphi )} \\ \end{array} } & {V_{\varphi } (\varphi )} & {M_{\varphi } (\varphi )]^{T} } \\ \end{array} $$

It is very difficult to directly determine the state vector of the bellows in the air spring, so that the intermediate vector of displacement *ξ* is introduced^22^. The intermediate vector of displacement consists of the bellows displacement and its corresponding partial derivative. It is expressed as:

7 $$ \xi (\varphi ) = {\left[\begin{array}{*{20}c} {u(\varphi )} & {v(\varphi )} & {w(\varphi )} & {\frac{\partial u(\varphi )}{{\partial \varphi }}}   & {\frac{\partial v(\varphi )}{{\partial \varphi }}} & {\frac{\partial w(\varphi )}{{\partial \varphi }}} & {\frac{{\partial^{2} w(\varphi )}}{{\partial \varphi^{2} }}} & \frac{{\partial^{2} \theta_{\varphi } (\varphi )}}{{\partial \varphi^{2} }}\end{array}\right]}^{T} $$

The physical equations^20^ and geometrical equations^22^ are substituted into Eq. (4) to obtain the first-order differential equation for the intermediate vector of displacement as follows:

8 $$ \frac{d\xi (\varphi )}{{d\varphi }} = C(\varphi )\xi (\varphi ) $$

where *C*_*w*_(*φ*) is an eight-order coefficient matrix with the elements listed in the Appendix. The rotary shell is partitioned into *N* nodes. The precise integration method^23^ is used to determine the transfer of the intermediate vector of displacement between nodes as follows:

9 $$ \xi (\varphi_{i + 1} ) = \exp (C_{w} (\varphi_{i} ) \bullet (\varphi_{i + 1} - \varphi_{i} ))\xi (\varphi_{i} ) = T_{c} \xi (\varphi_{i} ) $$

The relationship between the state vector of the bellows and the intermediate vector of displacement as follows:

10 $$ Z(\varphi ) = Q(\varphi )\xi (\varphi ) $$

where *E*(*φ*) is the incidence matrix, which is an eight-order coefficient matrix. The elements of the matrix are listed in the Appendix. By substituting Eq. (10) into Eq. (9), the transfer of the state vector of the bellows between nodes in the rotary shell can be obtained as follows:

11 $$ Z(\varphi_{i + 1} ) = Q(\varphi_{i + 1} )T_{C} (\varphi_{i} )Q^{ - 1} (\varphi_{i} )Z(\varphi_{i} ) = T_{i} Z(\varphi_{i} ) $$

### Determining the stiffness characteristics of an air spring

#### Solving the state vector of the bellows

Assume that the starting point of the circular bellows is *E* and the ending point is *F*. The state vector at the two points is:

12 $$ Z_{E} = \begin{array}{*{20}c} {\begin{array}{*{20}c} {\begin{array}{*{20}c} {\begin{array}{*{20}c} {[u_{E} } & {v_{E} } \\ \end{array} } & {w_{E} } & {\theta_{{\varphi_{E} }} } \\ \end{array} } & {N_{{\varphi_{E} }} } & {S_{{\varphi_{E} }} } \\ \end{array} } & {V_{{\varphi_{E} }} } & {M_{{\varphi_{E} }} ]^{T} } \\ \end{array} ,\begin{array}{*{20}c} {} \\ \end{array} Z_{F} = \begin{array}{*{20}c} {\begin{array}{*{20}c} {\begin{array}{*{20}c} {\begin{array}{*{20}c} {[u_{F} } & {v_{F} } \\ \end{array} } & {w_{F} } & {\theta_{{\varphi_{F} }} } \\ \end{array} } & {N_{{\varphi_{F} }} } & {S_{{\varphi_{F} }} } \\ \end{array} } & {V_{{\varphi_{F} }} } & {M_{{\varphi_{F} }} ]^{T} } \\ \end{array} $$

In order to solve all the state vectors of the circular bellows, the boundary conditions at both ends of the bellows must be specified. During operation, the air spring has its top mount plate connected to equipment. Thus it bears the equipment load, and is deformed with the motion of equipment. For this reason, the head of the bellows in the air spring is a free end. Assuming that the axial displacement of the top mount plate with equipment is *x* and its radial displacement is 0, the boundary conditions of the head of the bellows are defined by:

13 $$ \left\{ {\begin{array}{*{20}l} {u_{E} \cos \left( \varphi \right) + w_{E} \sin \left( \varphi \right)\left| {_{\varphi = \alpha } } \right. = 0,\;v_{E} \left| {_{\varphi = 2\pi - \beta } } \right. = 0} \hfill \\ {u_{E} \sin \left( \varphi \right) - w_{E} \cos \left( \varphi \right)\left| {_{\varphi = \alpha } } \right. = {\text{x,}}\;M_{{\varphi_{E} }} \left| {_{\varphi = \alpha } } \right. = 0} \hfill \\ \end{array} } \right. $$

The tail of the bellows is attached to the base, so that the boundary conditions at the tail of the bellows are given by:

14 $$ \left\{ \begin{gathered} u_{F} \left| {_{\varphi = 2\pi - \beta } } \right. = 0,\begin{array}{*{20}c} {\begin{array}{*{20}c} {} \\ \end{array} w_{F} \left| {_{\varphi = 2\pi - \beta } } \right. = 0} \\ \end{array} \hfill \\ v_{F} \left| {_{\varphi = 2\pi - \beta } } \right. = 0,\begin{array}{*{20}c} {} \\ \end{array} \theta_{{\varphi_{F} }} \left| {_{\varphi = 2\pi - \beta } } \right. = 0 \hfill \\ \end{gathered} \right. $$

Equations (13) and (14) are substituted into Eq. (11) to solve the state vector at the nodes of the bellows.

#### Solving the stiffness characteristics of the air spring

The stiffness of the air spring is the aggregate variation of the reaction forces of the air and bellows under unit displacement. It is assumed that *K*_*Z*_ is the total stiffness of the air spring, *K*_*Q*_ is the pneumatic stiffness, and *K*_*N*_ is the bellows stiffness. The stiffness of the air spring is expressed as:

15 $$ K_{Z} = K_{Q} + K_{N} $$

There is already a specific analytic solution for pneumatic stiffness *K*_*Q*_ of the air spring^24^, so that it is not detailed in this paper. The bellows stiffness of the air spring will be particularly solved in this paper. Since the state vector at the head of the bellows is known, the force analysis is conducted at the head of the bellows in the air spring. The reaction force of the bellows *F*_*N*_ is expressed as:

16 $$ F_{N} = (N_{{\varphi_{E} }} + S_{{\varphi_{E} }} )*2\pi R_{{\theta_{E} }}^{{}} $$

The internal air pressure and the state vector of capslue also vary with deformation. The total deformation of the air spring is decomposed into *n* small deformations. Every small deformation of the air spring is subject to the following assumptions:

1. The arc segment of the bellows is always arched in the process of deformation;
2. The change of internal air pressure complies with the adiabatic equation in the process of deformation;
3. The internal air pressure *P,* the structural parameters *R*_*e*_ and *R*_*φ*_ in each small deformation remains unchanged.

When the air spring is in the *i*th small deformation, the displacement at the head of its bellows changes from *x*_*i*-1_ to *x*_*i*_. Based on the above assumptions (1), (2) and (3), the structural parameters *R*_*e*_ and *R*_*φ*_ and the internal air pressure *P*_*i*_ of the air spring in the *i*th small deformation are given by:

17 $$ \left\{ {\begin{array}{*{20}l} {R_{e}^{i} { = }R_{{\text{e}}}^{{i{ - 1}}} { + }A_{\text{R}} (x_{i} - x_{i - 1} )} \hfill \\ {R_{\varphi }^{i} { = }R_{\varphi }^{{i{ - 1}}} { + }A_{{{\text{R}}_{\varphi } }} (x_{i} - x_{i - 1} )} \hfill \\ {A_{{\text{Re}}} = \frac{{(3\pi /2 - \beta ){\text{cos}}\alpha {\text{cos}}\beta + sin(\pi /2 + \beta )}}{2[1 - cos(\pi /2 + \beta )] + (3\pi /2 - \beta )sin(\pi /2 + \beta )}} \hfill \\ {A_{{{\text{R}}_{\varphi } }} = - \frac{{{\text{cos}}\beta }}{(2(1 - cos(\beta + \pi /2)) + (3\pi /2 - \beta )(sin(\beta + \pi /2))}} \hfill \\ {P_{i} = (P_{a} + P_{i - 1} )V_{i - 1}^{n} /V_{i}^{n} - P_{a} } \hfill \\ \end{array} } \right. $$

In Eq. (17),* R*_*e*_ is the effective radius; *R*_*φ*_ is the bellows radius; *P*_*a*_ is the atmosphere pressure; *P*_*i*-1_ and *V*_*i*-1_ are the air pressure and capacity of the air spring under the displacement *x*_*i*−1_; *P*_*i*_ and *V*_*i*_ are the air pressure and capacity of the air spring under the axial displacement *x*_*i*_; *n* is the polytropic coefficient;

When the air pressure and structural parameters of the bellows at the *i*th small deformation are known, Eqs. (11), (13), (14) and (16) are used to determine the variation of the reaction force of the bellows at the *i*th small deformation, that is, *ΔF*_*Ni*_. The bellows stiffness during the deformation can be calculated through iteration as follows:

18 $$ K_{N} = \left( {\sum\limits_{i = 1}^{n} {\Delta F_{Ni} } } \right)/x $$

## Test verification and analysis

**Figure 3:**
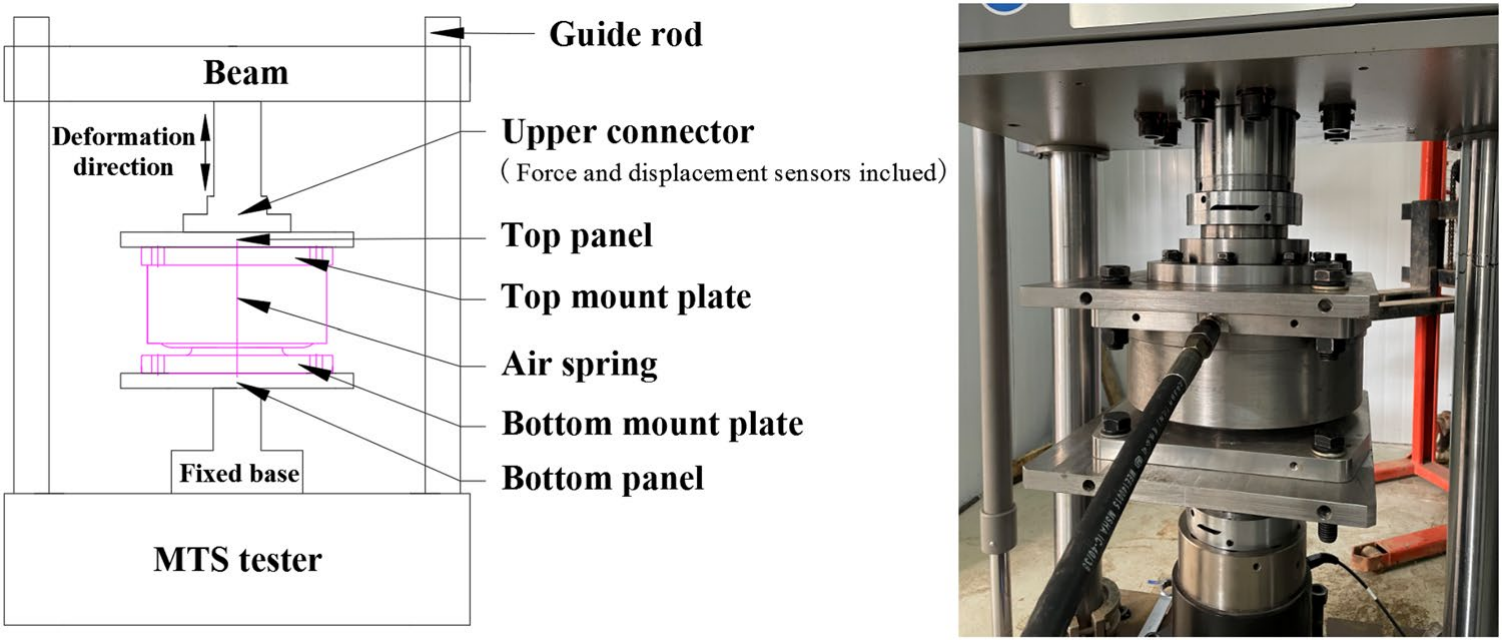
Schematic diagram of the installation structure in the stiffness test of an air spring.

A stiffness test was carried out for air springs installed as shown in Fig. 3. The top mount plate of each air spring was connected through its top panel to the upper connector of the MTS tester. The upper connector was equipped with the force and displacement sensors. The bottom mount plate was attached through its bottom panel to the fixed base. During the stiffness test of each air spring, the top mount plate moved back and forth with the upper connector, while the bottom mount plate was stationary. The readings of the force and displacement sensor were output from the computer to calculate the stiffness of each air spring.

### Test verification

Static and dynamic stiffness tests were conducted on M-8T, M-15T and M-30T air springs. For marine air springs, the displacement peak-to-peak value is set to 1 mm, and the displacement rate is set to 0.1 mm/s in the static stiffness test. In the dynamic test, the displacement peak-to-peak value is set to 0.4 mm and the excitation frequency is set to 2.5 Hz. Two prototypes were selected for each type of air spring in the stiffness test, and their average value was taken after the test. It should be noted that the solution method for the bellows stiffness in this paper does not specifically calculate the static or the dynamic stiffness, but the existing pneumatic stiffness theory model can calculate the static and the dynamic stiffness. The static stiffness is denoted by $K_{{}}^{s}$, and the dynamic stiffness is denoted by $K_{{}}^{d}$.

The main design parameters of these air springs are detailed in Table 1, and the material parameters of the cord are detailed in Table 2. The theoretical calculation and test results of their stiffness characteristics are listed in Table 3. As given in Table 3, the error of stiffness test for these air springs was within 10%, which verifies the accuracy of the theoretical model for the stiffness of the air springs. Additionally, the stiffness of the bellows has been considerably improved with the greatly reinforced structure of the bellows in an air spring. The bellows stiffness of M-8T air spring is higher than its pneumatic stiffness. Among the three types of air spring, the lowest ratio of the bellows stiffness to the total stiffness is 26.7%. The influence of the bellows own on the stiffness characteristics of air springs is not negligible.

**Table 1:** Main design parameters of air springs.

M-8T air spring		M-15T air spring		M-30T air spring	
Item	Value	Item	Value	Item	Value
Effective radius $R_{\varphi }$	26 mm	Effective radius $R_{\varphi }$	22.5 mm	Effective radius $R_{\varphi }$	32.5 mm
Bellows radius $R_{e}$	108 mm	Bellows radius $R_{e}$	141 mm	Bellows radius $R_{e}$	195 mm
Guiding angle $\beta$	90°	Guiding angle $\beta$	54°	Guiding angle $\beta$	60°
Cord layer thickness $h$	6.6 mm	Cord layer thickness $h$	4.4 mm	Cord layer thickness $h$	8.8 mm
Initial filament winding angle *δ*	28.9°	Initial filament winding angle *δ*	24.8°	Initial filament winding angle *δ*	27.2°

**Table 2:** Cord material parameters.

Item	Value	Item	Value	Item	Value	Item	Value
Elastic modulus *E*_1_^20^	49.7 GPa	Elastic modulus *E*_2_^20^	6 MPa	ShearModulus *G*_12_^20^	2.5 MPa	Poisson ratio *v*_21_^20^	0.45

**Table 3:** Theoretical calculation and test result.

Item	M-8T air spring	M-15T air spring	M-30T air spring
Theoretical calculation (kN/mm)			
$K_{N}$	1.21	1.73	2.26
$K_{Q}^{s}$	0.85	3.14	4.91
$K_{Q}^{d}$	1.20	3.96	6.19
$K_{Z}^{s}$	2.06	4.87	7.17
$K_{Z}^{d}$	2.41	5.69	8.45
Test result (kN/mm)			
$K_{Z}^{s}$	2.22	4.98	7.39
$K_{Z}^{d}$	2.57	5.51	8.01
Error of theoretical calculation			
$K_{Z}^{s}$	7.21%	2.21%	2.98%
$K_{Z}^{d}$	6.23%	3.27%	5.49%

### Performance analysis

The bellows stiffness model is the main research of this paper. The pneumatic model now has a clear analytical solution, and different design parameters have a similar trend of influence on static and dynamic pneumatic stiffness. In addition, considering the cord-reinforced air spring are used in marine vibration isolation systems, and the study of static stiffness is of great importance for the overall attitude control of marine vibration isolation systems. Therefore, static stiffness was used as a research objective, and the structural parameters of M-15T air spring are taken as the basis to analyze the variation law of the stiffness characteristics under different conditions including preload, structural parameters, and material parameters.

#### Influence of the preload on the stiffness characteristics of the air spring

**Figure 4:**
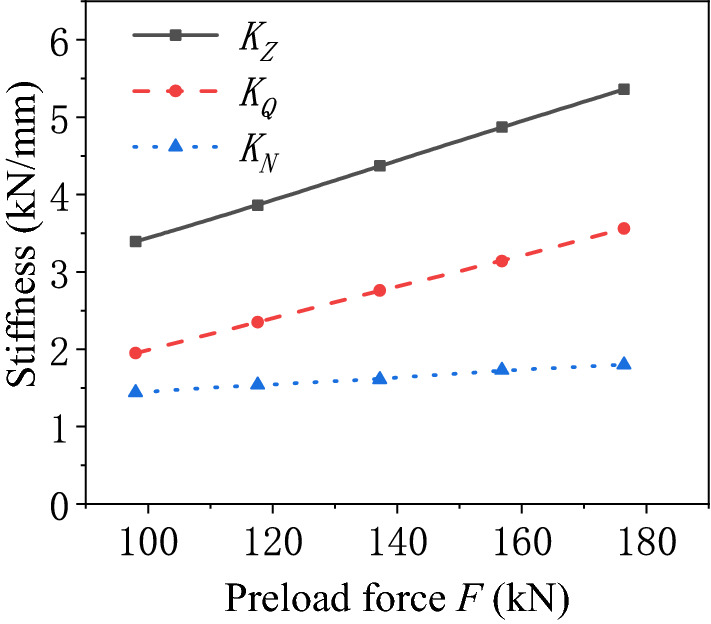
Schematic diagram of the stiffness of the air spring varying with the preload force.

The stiffness characteristics of the air spring under different preloads are calculated as given in Fig. 4. The pneumatic stiffness and bellows stiffness of the air spring improve with the increasing preload. Their relationship is approximately linear. During the variation of the preload, the pneumatic stiffness is more severely affected by the preload. Hence, the pneumatic stiffness dominates the trend of the total stiffness of the air spring. The total stiffness is linearly related to the variation of the preload.

#### Influence of the geometrical structural parameters on the stiffness characteristics of the air spring

1. Influence of the effective radius of the air spring.
  The stiffness characteristics of the air spring are calculated after changing the effective radius of the air spring as illustrated in Fig. 5. Evidently, the pneumatic stiffness and bellows stiffness of the air spring improve with the increasing effective radius of the air spring. The bellows stiffness varies approximately linearly with the effective radius. The variation of the pneumatic stiffness with the effective radius is approximate to a quadratic function. The pneumatic stiffness is more significantly affected by the variation of effective radius, so that it dominates the variation trend of the total stiffness of the air spring. Consequently, the variation of the total stiffness with the effective radius is approximate to a quadratic function. When the effective radius of the air spring is large, the pneumatic stiffness of the air spring is greater than its bellows stiffness. With the decrease of the effective radius, the proportion of bellows stiffness in total stiffness gradually increases until it exceeds the contribution of the pneumatic stiffness. By then, the stiffness characteristics of the air spring will depend on the bellows stiffness.

**Figure 5:**
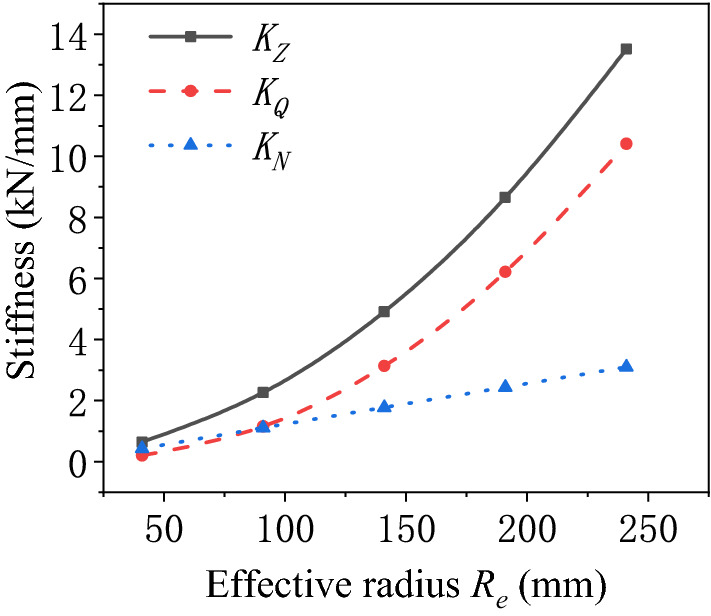
Schematic diagram of the stiffness of the air spring varying with the effective radius.
2. Influence of the bellows radius of the air spring.
  The stiffness characteristics of the air spring are calculated with the varying bellows radius of the air spring as shown in Fig. 6. It is revealed that the pneumatic stiffness and bellows stiffness of the air spring decrease with the increasing bellows radius of the air spring. The variation of the bellows stiffness with the bellows radius is approximately a quadratic function, while the variation of the pneumatic stiffness with the bellows radius is approximately linear. The bellows stiffness is more significantly affected by the variation of the bellows radius, so that it makes the principal contribution to the variation of the total stiffness of the air spring. The variation of the total stiffness with the bellows radius is approximately a quadratic function. When the bellows radius of the air spring is large, the air spring has pneumatic stiffness greater than bellows stiffness. However, the decreasing bellows radius gradually pushes up the contribution of the bellows stiffness to the total stiffness of the air spring. In the end, the bellows stiffness surpasses the pneumatic stiffness in terms of the contribution to the total stiffness. At that time, the stiffness characteristics of the air spring will depend on the bellows stiffness.

**Figure 6:**
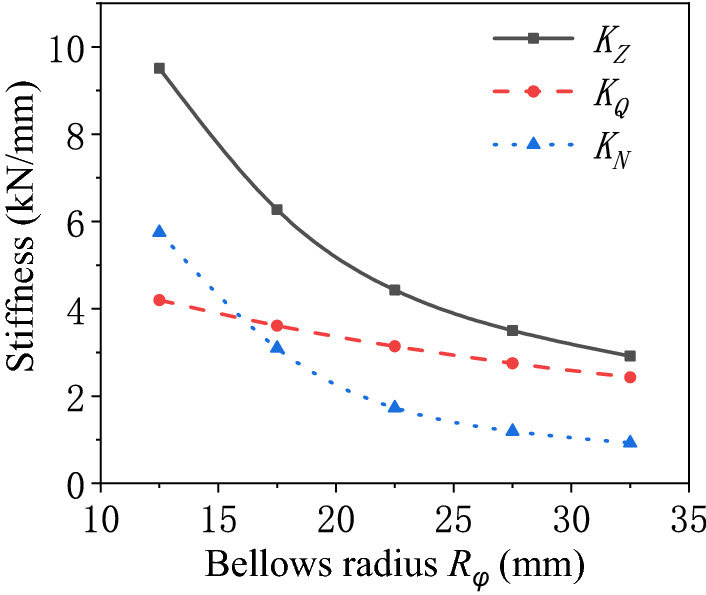
Schematic diagram of the stiffness of the air spring varying with the bellows radius.
3. Influence of the guiding angle of the air spring.
  The stiffness of the air spring is calculated with different guiding angles as shown in Fig. 7. The guiding angle of the air spring increases, which causes the decrease of the pneumatic stiffness and bellows stiffness of the air spring. The variation of the pneumatic stiffness with the guiding angle is approximately a quadratic function. There is approximately a linear relationship of variation between the bellows stiffness and the guiding angle. The pneumatic stiffness is more strongly affected by the variation of the guiding angle, and is always greater than the bellows stiffness. The variation of the total stiffness of the air spring is dominated by the pneumatic stiffness. Hence, the variation of the total stiffness with the guiding angle is approximately a quadratic function.

**Figure 7:**
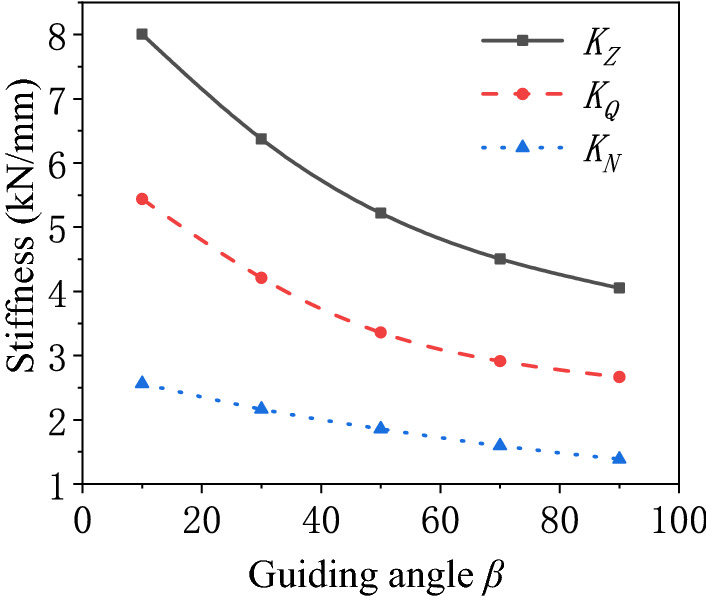
Schematic diagram of the stiffness of the air spring varying with the guiding angle.

#### Influence of the material characteristics parameters on the stiffness characteristics of the air spring

1. Influence of the initial winding angle on the stiffness characteristics of the air spring.
  The initial winding angle is a crucial parameter to the fabrication of the bellows. It decides the cord winding angle at each point of the bellows^20^. The stiffness characteristics of the air spring with different initial winding angles are calculated as presented in Fig. 8. As revealed, the pneumatic stiffness of the air spring is not affected by the variation of the initial winding angle. The bellows stiffness improves and then declines with the increase of the initial winding angle. It peaks when the initial winding angle is 25.2°. On the whole, the maximum variation of bellows stiffness is only 4.1% of the total stiffness during the variation of the initial winding angle. It is therefore concluded that the initial winding angle exerts a little effect on the stiffness characteristics of the air spring.

**Figure 8:**
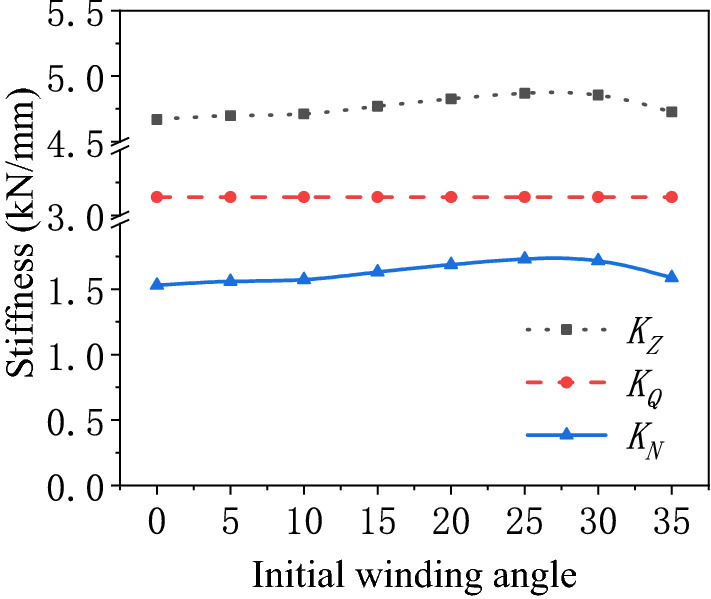
Schematic diagram of the stiffness of the air spring varying with the initial winding angle.
2. Influence of the cord layer structural parameter on the stiffness characteristics.
  The elastic modulus of the cord in the main direction* E*_*1*_ is much larger than that in the other directions. Therefore, this paper mainly focuses on the influence of the elastic modulus of the cord* E*_*1*_ on the stiffness characteristics of the air spring. In the theoretical calculation, it is found that the bellows stiffness of the air spring varies approximately linearly with the elastic modulus of the cord *E*_*1*_ and the thickness of the cord layer* h*. The product of the above elastic modulus *E*_*1*_ and thickness* h* is taken as the cord layer structural parameter*γ* to analyze the influence of the cord layer structural parameter on the bellows stiffness of the air spring. After changing the scale-down of the cord layer structural parameter in the air spring, the stiffness characteristics of the air spring with different scale-downs are calculated as shown in Fig. 9. The pneumatic stiffness is not affected by the variation of the cord layer structural parameter, but the bellows stiffness improves with the increase of the cord layer structural parameter.

**Figure 9:**
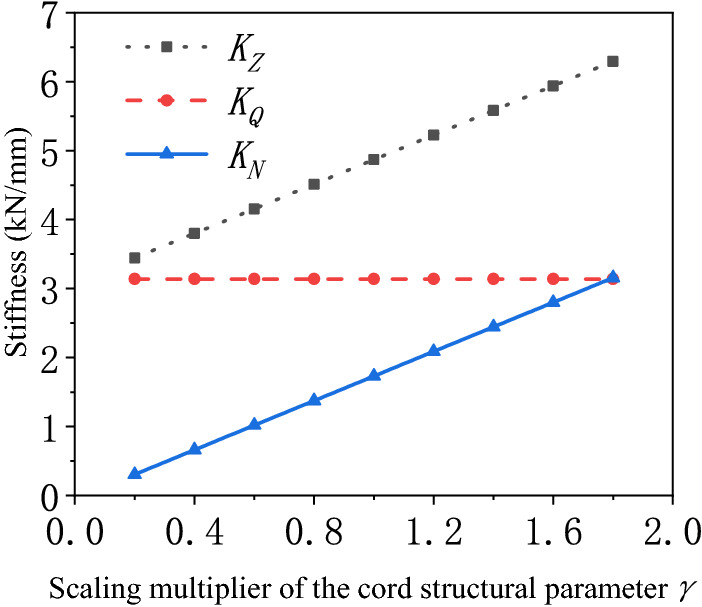
Scale-down variation of the stiffness of the air spring with the cord layer structural parameter.
  The comparison of Figs. 8 and 9 reveals that the cord layer structural parameter is the main factor affecting the bellows stiffness of the air spring. The stiffness of the air spring contains bellows stiffness and pneumatic stiffness. It is assumed that the bellows stiffness may be ignored while calculating the stiffness of the air spring only if the bellows stiffness is less than 10% of the total stiffness. The calculation exposes that the ratio of the bellows stiffness to the total stiffness of the air spring varies with the cord layer structural parameter as shown in Fig. 10. The cord layer structural parameter may be adjusted by changing the type of cord or the cord winding thickness. When the cord structure parameter is adjusted and lowered by 0.23 times, the influence of the bellows stiffness on the total stiffness of the air spring is negligible.

**Figure 10:**
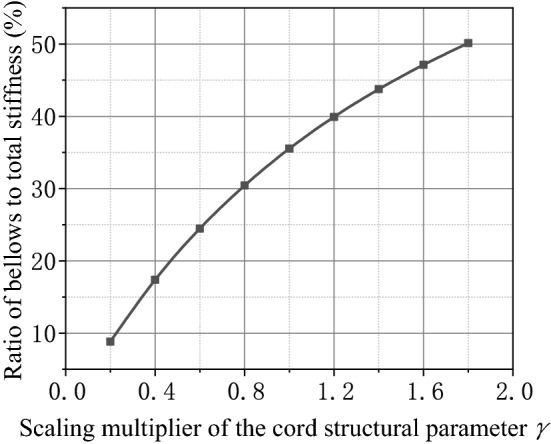
Schematic diagram of the ratio of the bellows stiffness to the total stiffness varying with the scale-down of the cord layer structural parameter.

## Conclusion

With the precise transfer integration method, a parameterized model is built for the bellows stiffness of the cord-reinforced air spring with winding formation under preload conditions. Moreover, the influence of the bellows stiffness and the pneumatic stiffness on the stiffness characteristics of the air spring is analyzed. After exploring the influence of different preloads, geometrical structural parameters, and bellows material characteristics on the stiffness characteristics of the air spring, the following conclusions are drawn:

1. The bellows stiffness and pneumatic stiffness of the cord-reinforced air spring improve with the increase of preload, but decline with the increase of guiding angle. There is a linear relationship between the bellows stiffness and pneumatic stiffness of the air spring and the variation of preload. The variation of the pneumatic stiffness with the guiding angle forms a quadratic function, while the variation of the bellows stiffness with the guiding angle is linear. The pneumatic stiffness is more strongly affected by the variation of preload and guiding angle. The variation of the total stiffness of the air spring is dominated by the pneumatic stiffness, and is basically consistent with the variation of the pneumatic stiffness with the preload and guiding angle.
2. The bellows and pneumatic stiffness of the cord-reinforced air spring heighten with the increase of effective radius, but decrease with the increase of bellows radius. The variation of the pneumatic stiffness with the effective radius is represented by a quadratic function, but the variation of the pneumatic stiffness with bellows radius is linear. The pneumatic stiffness is more strongly affected by the variation of effective radius, and therefore makes the principal contribution to the varying total stiffness of the air spring. However, the pneumatic stiffness varies linearly with the bellows radius, while a quadratic function exists for the variation of the bellows stiffness with bellows radius. For this reason, the bellows stiffness is more strongly affected by the variation of bellows radius, and therefore dominates the variation trend of the total stiffness of the air spring. When the effective radius or bellows radius is large, the pneumatic stiffness of the air spring is greater than the bellows stiffness. Along with the decrease of the effective radius or bellows radius, the contribution of the bellows stiffness to the total stiffness of the air spring improves gradually until the bellows stiffness surpasses the pneumatic stiffness. At this time, the stiffness characteristics of the air spring depend on the bellows stiffness.
3. The bellows stiffness of an air spring improves with the increase of cord layer structural parameter, but goes up and then down with the increase of initial winding angle. On the contrary, the pneumatic stiffness is not affected by cord layer structural parameter or initial winding angle. The initial winding angle exerts a lower effect on the bellows stiffness than the cord layer structural parameter. Therefore, the influence of the bellows stiffness on the stiffness characteristics of the air spring can be effectively reduced by lowering the cord winding thickness or changing to a cord of lower elastic modulus.

## Supplementary Information

Supplementary Information.

## Data Availability

The data used to support the findings of this study are available from the corresponding author upon request.
