# Supplementary material for: Bellows stiffness characteristics of cord-reinforced air spring with winding formation under preload conditions
Source: Sci Rep. 2023 Feb 28;13:3377. doi: 10.1038/s41598-023-29474-3 (PMC9974950; doi:10.1038/s41598-023-29474-3)
Supplement: Supplementary file 1 — Supplementary Information. [file 41598_2023_29474_MOESM1_ESM.docx]

**Appendix** Coefficient Matrix Elements

(1) Elements in the matrix *Q*(*ϕ*)

, , , , , ,

, ,

, , , , , ,

(2) Elements in the matrix *C*(*ϕ*)
